# Bilateral Cerebellar Infarction Caused by Occlusion of a Bihemispheric Posterior Inferior Cerebellar Artery Variant: A Case Report

**DOI:** 10.7759/cureus.80496

**Published:** 2025-03-12

**Authors:** Katsuya Nishida, Kento Sakashita

**Affiliations:** 1 Neurology, National Hospital Organization Hyogo Chuo National Hospital, Sanda, JPN

**Keywords:** bihemispheric pica variant, bilateral cerebellar infarction, hydrocephalus, magnetic resonance angiography (mra), posterior inferior cerebellar artery, stroke

## Abstract

Bilateral cerebellar infarction due to unilateral occlusion of the posterior inferior cerebellar artery (PICA) is extremely rare, particularly when a bihemispheric PICA variant is present. We report the case of an 81-year-old male with a history of hypertension and diabetes who presented with recurrent vomiting and gait disturbance, eventually progressing to a comatose state. MRI revealed bilateral cerebellar infarctions sparing the brainstem and ventricular enlargement consistent with obstructive hydrocephalus. Magnetic resonance angiography (MRA) demonstrated occlusion of the right PICA, while an earlier MRA had identified a bihemispheric PICA variant originating from the right vertebral artery and supplying both cerebellar hemispheres. Echocardiography did not reveal any embolic source. This case underscores the clinical importance of early recognition of rare vascular anomalies, such as the bihemispheric PICA variant, which is critical for optimal stroke management and the prevention of complications like obstructive hydrocephalus.

## Introduction

Bilateral cerebellar infarction is a rare yet severe condition, typically resulting from simultaneous occlusion of both cerebellar arteries. However, in exceptional cases, it may arise due to unique anatomical variations such as the bihemispheric posterior inferior cerebellar artery (PICA) variant. This variant, characterized by a single artery supplying both cerebellar hemispheres, significantly increases the risk of bilateral infarction even with unilateral vessel occlusion [1-8]. We present a case where this anomaly was identified early through imaging, discussing its clinical implications and management strategies.

## Case presentation

**Figure 1:**
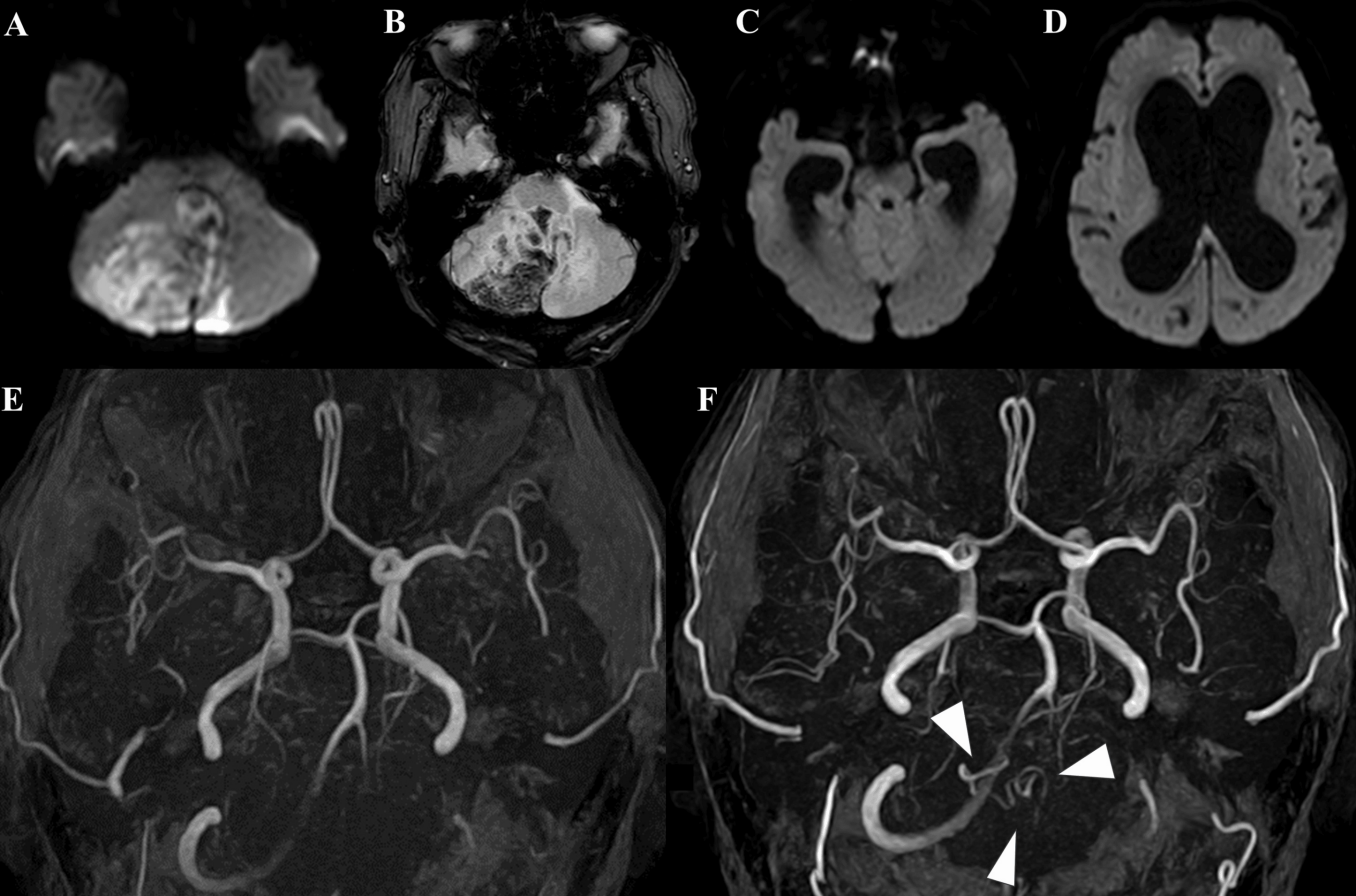
MRI and MRA in a patient with bilateral PICA infarction (A) DWI shows bilateral cerebellar infarctions sparing the brainstem. (B) T2*-weighted imaging shows low signal intensity in the infarcted areas. (C, D) DWI shows ventricular enlargement, consistent with obstructive hydrocephalus. (E) MRA from the current presentation shows occlusion of the right PICA. (F) MRA performed two years earlier reveals a bihemispheric PICA variant arising from the right vertebral artery, while the contralateral PICA was not visualized (arrowhead indicates the right PICA). DWI, diffusion-weighted imaging; MRA, magnetic resonance angiography; PICA, posterior inferior cerebellar artery

An 81-year-old male with a history of hypertension and diabetes managed with biguanide-based medication presented with recurrent vomiting and gait disturbance. Over several days, his condition deteriorated rapidly, culminating in a comatose state. MRI revealed bilateral hemorrhagic cerebellar infarctions sparing the brainstem and ventricular enlargement consistent with obstructive hydrocephalus (Figure 1A-1D).

Due to the patient’s treatment with biguanide-based medication, a CT angiogram was not performed on admission; instead, magnetic resonance angiography (MRA) was used for vascular evaluation. The acute-phase MRA revealed occlusion of the right PICA (Figure 1E), while an MRA from two years earlier had shown a bihemispheric PICA variant arising from the right vertebral artery (Figure 1F). The left vertebral artery was hypoplastic, and the left PICA was not visualized.

An ECG on admission and continuous ECG monitoring over several days did not reveal any atrial fibrillation. Furthermore, echocardiography demonstrated normal left ventricular wall motion with no evidence of atrial enlargement or intracardiac thrombus, suggesting that the stroke was more likely due to an atherosclerotic mechanism rather than a cardioembolic source.

The patient and his family declined surgical or invasive treatments; therefore, conservative medical management was continued. Over time, his level of consciousness improved, and he became capable of oral intake, although severe gait disturbance persisted. He was subsequently transferred to a rehabilitation hospital for further management.

## Discussion

Unilateral PICA occlusion causing bilateral cerebellar infarction is rare but carries significant clinical implications. The bihemispheric PICA variant markedly increases the risk of bilateral infarction even when only one vessel is occluded [1,5,8]. This case highlights the importance of recognizing vascular anomalies to optimize treatment strategies and improve outcomes. MRA, as demonstrated here, is particularly useful for identifying such anomalies before clinical symptoms appear [2,6].

The PICA is one of the most anatomically variable arteries in the brain [1,5]. While digital subtraction angiography traditionally provides detailed visualization of these variants, MRA was effective in clearly demonstrating the bihemispheric configuration in this case [5,8]. Detection of this vascular anomaly two years prior to stroke underscores the value of routine imaging in identifying high-risk anatomical variants, even in asymptomatic patients [8]. Early detection could facilitate proactive management strategies, potentially mitigating severe outcomes [7].

A previous study has reported that cerebellar infarctions account for 2.3% of all brain infarctions, with approximately half occurring in the PICA territory, and that bilateral cerebellar infarctions constitute 12% of cerebellar strokes [9]. Another study demonstrated that 31% of cerebellar infarctions are bilateral and that these bilateral infarcts are associated with more severe initial clinical symptoms, a more unstable hospital course, and poorer outcomes at discharge compared to unilateral infarctions [10]. These findings suggest that vascular anomalies, particularly the bihemispheric PICA variant, may predispose patients to bilateral cerebellar infarctions and contribute significantly to their poorer clinical outcomes.

When comparing infarcts in different cerebellar arterial territories, Kim et al. [11] found that bilateral infarctions in the superior cerebellar artery (SCA) territory were predominantly associated with cardioembolic sources, whereas infarctions in the PICA territory, as observed in our case, are more often linked to an atherosclerotic mechanism [10]. This contrast underscores that, although bilateral infarcts can occur in both SCA and PICA territories, the underlying etiologies differ. As a result, early detection of a bihemispheric PICA variant may justify more aggressive management of atherosclerotic risk factors, such as tighter control of hypertension and diabetes, and the initiation of antiplatelet therapy. Tailoring preventive strategies based on the stroke mechanism could ultimately improve patient outcomes.

In the acute phase of stroke evaluation, MRA plays a crucial role not only in confirming the diagnosis but also in identifying the underlying stroke mechanism. In our case, the acute-phase MRA findings suggested an atherosclerotic etiology, which influenced our decision to pursue antiplatelet therapy as part of the management strategy. Therefore, the use of MRA during the acute phase provides essential information that goes beyond simply detecting vascular anomalies, enabling tailored treatment strategies for individual patients.

Carlson et al. [8] reported that the true incidence of bihemispheric PICA variants may be much higher (approximately 3-4%) than previously estimated (<0.1% [5]), reinforcing their clinical relevance in ischemic stroke management. Furthermore, large cerebellar infarctions often lead to obstructive hydrocephalus due to brainstem compression and impaired cerebrospinal fluid flow [1,4]. Recognizing these complications promptly is crucial for appropriate intervention, as demonstrated in our case [1,2,4].

## Conclusions

Early identification of vascular anomalies, including the bihemispheric PICA variant, is essential for optimal stroke management. This case illustrates how rare anatomical variations significantly influence cerebrovascular events, emphasizing the necessity for thorough vascular assessment in stroke-prone patients. Incorporating routine imaging techniques such as MRA into clinical practice could facilitate the timely detection of these anomalies, guiding appropriate treatment decisions and preventing severe complications like obstructive hydrocephalus. This report underscores the importance of raising clinicians’ awareness of rare vascular anomalies to optimize patient outcomes.
